# The Italian Society for Pediatric Nephrology (SINePe) consensus document on the management of nephrotic syndrome in children: Part I - Diagnosis and treatment of the first episode and the first relapse

**DOI:** 10.1186/s13052-017-0356-x

**Published:** 2017-04-21

**Authors:** Andrea Pasini, Elisa Benetti, Giovanni Conti, Luciana Ghio, Marta Lepore, Laura Massella, Daniela Molino, Licia Peruzzi, Francesco Emma, Carmelo Fede, Antonella Trivelli, Silvio Maringhini, Marco Materassi, Giovanni Messina, Giovanni Montini, Luisa Murer, Carmine Pecoraro, Marco Pennesi

**Affiliations:** 1 Nephrology and Dialysis Unit, Department of Pediatrics, Azienda Ospedaliero Universitaria, Policlinico Sant’Orsola-Malpighi, Bologna, Italy; 2 Pediatric Nephrology, Dialysis and Transplant Unit, Department of Pediatrics, University Hospital of Padua, Padua, Italy; 3 Pediatric Nephrology and Rheumatology Unit with Dialysis, AOU G. Martino, Messina, Italy; 4 Pediatric Nephrology and Dialysis Unit, Fondazione Ca’ Granda, IRCCS Ospedale Maggiore, Policlinico Milano, Milan, Italy; 5 Nephrology and Dialysis Unit, Pediatric Subspecialties Department, Bambino Gesù Children’s Hospital, IRCCS, Rome, Italy; 6 Santobono Children’s Hospital, Naples, Italy; 7 City of the Health and the Science of Turin Health Agency, Regina Margherita Children’s Hospital, Turin, Italy; 8 Division of Nephrology, Dialysis, Transplantation, and Laboratory on Pathophysiology of Uremia, Istituto G. Gaslini, Genoa, Italy; 9 Pediatric Nephrology Unit, Children’s Hospital ‘G. Di Cristina’, A.R.N.A.S. ‘Civico’, Palermo, Italy; 10 Nephrology and Dialysis Unit, Meyer Children’s Hospital, Florence, Italy; 11 Nephrology Unit, Giovanni XXIII Children’s Hospital, Bari, Italy; 12 Institute of Maternal and Child Health IRCCS “Burlo Garofolo”, Department of Pediatrics, Trieste, Italy

**Keywords:** Nephrotic syndrome, Prednisolone, Steroid sensitive, Steroid resistant, Edema, Diuretics, Thromboembolism, Anticoagulation agents, Kidney biopsy

## Abstract

This consensus document is aimed at providing an updated, multidisciplinary overview on the diagnosis and treatment of pediatric nephrotic syndrome (NS) at first presentation. It is the first consensus document of its kind to be produced by all the pediatric nephrology centres in Italy, in line with what is already present in other countries such as France, Germany and the USA. It is based on the current knowledge surrounding the symptomatic and steroid treatment of NS, with a view to providing the basis for a separate consensus document on the treatment of relapses. NS is one of the most common pediatric glomerular diseases, with an incidence of around 2–7 cases per 100000 children per year. Corticosteroids are the mainstay of treatment, but the optimal therapeutic regimen for managing childhood idiopathic NS is still under debate. In Italy, shared treatment guidelines were lacking and, consequently, the choice of steroid regimen was based on the clinical expertise of each individual unit. On the basis of the 2015 Cochrane systematic review, KDIGO Guidelines and more recent data from the literature, this working group, with the contribution of all the pediatric nephrology centres in Italy and on the behalf of the Italian Society of Pediatric Nephrology, has produced a shared steroid protocol that will be useful for National Health System hospitals and pediatricians. Investigations at initial presentation and the principal causes of NS to be screened are suggested. In the early phase of the disease, symptomatic treatment is also important as many severe complications can occur which are either directly related to the pathophysiology of the underlying NS or to the steroid treatment itself. To date, very few studies have been published on the prophylaxis and treatment of these early complications, while recommendations are either lacking or conflicting. This consensus provides indications for the prevention, early recognition and treatment of these complications (management of edema and hypovolemia, therapy and prophylaxis of infections and thromboembolic events). Finally, recommendations about the clinical definition of steroid resistance and its initial diagnostic management, as well as indications for renal biopsy are provided.

## Introduction

In Italy, shared treatment guidelines were lacking and, consequently, the choice of steroid regimen was based on the clinical expertise of each individual unit. This consensus document is aimed at providing an updated, multidisciplinary overview on the diagnosis and treatment of pediatric nephrotic syndrome (NS) at first presentation, proposing a shared steroid protocol for National Health System hospitals and pediatricians. Furthermore, indications for the prevention, early recognition and treatment of NS complications (management of edema and hypovolemia, therapy and prophylaxis of infections and thromboembolic events), as well as recommendations about the clinical definition of steroid resistance and its initial diagnostic management, and indications for renal biopsy are provided.

It is based on the current knowledge surrounding the symptomatic and steroid treatment of NS, with a view to providing the basis for a separate consensus document on the treatment of relapses and for future research. Before writing the document, the working group conducted a thorough review of the literature in the PubMed database up to August 2015.

## Background

Nephrotic syndrome is a rare disease with an incidence of around 2–7 cases per 100,000 children per year and a prevalence of nearly 16 cases per 100,000 [1]. The International Study of Kidney Disease in Childhood (ISKDC) determined the histopathological, clinical and laboratory characteristics of NS in children [2] and demonstrated that minimal change disease (MCD) accounts for 76% of idiopathic NS (INS) cases. Subjects with MCD have a 95% response rate to steroids, however, 75% will relapse and 50% (frequent relapsers or steroid dependent subjects) will require higher and prolonged doses of steroids thus increasing the risk of side effects [3]. In any case, in terms of renal function, response to steroids is associated with a good long-term prognosis.

## Diagnosis

Nephrotic syndrome is defined by the presence of [4]:

- Heavy proteinuria: ≥50 mg/kg/day (or ≥40 mg/m2/h), or a proteinuria/creatininuria ratio >2 (mg/mg)
- Serum albumin < 25 g/L
- Edema

During the initial assessment of a child with a first episode of NS, the aim of the pediatrician is to:

- Assess whether the NS is primary or secondary in nature [1] (Table 1); exclude other renal pathologies presenting with edema and/or hypoalbuminemia (acute and chronic glomerulonephritis, Hemolytic-Uremic Syndrome, chronic renal failure)
- Start adequate therapy as early as possible.

**Table 1:** Causes of nephrotic syndrome

Primary Nephrotic Syndrome (95% in children 0–12 years)	
Idiopathic nephrotic syndrome (80-90% in children 2–8 years)	
Steroid-sensitive nephrotic syndrome	
Steroid-resistant nephrotic syndrome	
Genetic nephrotic syndrome (isolated or syndromic)	
(95 -100% in children <3 months	
50 - 60% in children 4–12 months)	
Secondary Nephrotic Syndrome (5% in children 0–12 years)	
- Vasculitides/autoimmune diseases (SLE, Microscopic polyangiitis, Goodpasture, IgA vasculitis)	
- Infections (HBV, HCV, HIV, EBV, Mycoplasma, CMV, PVB19, Treponema, Toxoplasma, malaria, parasites)	
- Drugs (Tiopronin, Penicillamine, Gold Salts, Pamidronate, Interferon, Everolimus, antiretroviral and chemotherapy drugs)	
- Diabetes	
- Cancer (Lymphoma, Leukemia)	

## Investigations at initial presentation

Children with NS at onset should be admitted to hospital and undergo a complete clinical and laboratory workup (Tables 2, 3 and 4). Imaging examinations are generally not helpful and should be guided by specific clinical indications (e.g. chest X-ray in the case of pulmonary edema/infection, renal ultrasound to exclude a rare condition of leukemic infiltration, etc.).

**Table 2:** Medical history

History	Family	General	Past	Current
Questions	NS in the family	Pre/peri-natal history	Systemic diseases (autoimmune, neurological, metabolic, congenital, cancer)	Timing and characteristics of edema
	Other kidney diseases in the family	Growth	Past infections	Associated signs/symptoms (macro/microscopic hematuria, fever, oliguria, vomiting, abdominal pain, hypertension, skin rash, arthralgia…),
	Other diseases in the family	Age at onset of symptoms		Travel/infections
				Drugs/poisons

**Table 3:** Physical examination

Clinical parameters	Edema	Signs/symptoms of hypovolemia	Signs/symptoms of infectious/systemic disease
▪ Heart rate ▪ Respiratory rate ▪ Blood pressure ▪ O2 saturation ▪ Body weight	▪ Periorbital ▪ Pretibial ▪ Genital ▪ Ascites ▪ Bowel wall edema ▪ Pleural effusion ▪ Pulmonary edema ▪ Anasarca	▪ Abdominal pain ▪ Tachycardia ▪ Cold hands/feet ▪ Oliguria ▪ Capillary refill >2 s	▪ Fever ▪ Skin rash ▪ Purpura ▪ Arthritis

**Table 4:** Biochemical tests

Tests	Blood	Urine
Mandatory	▪ Complete Blood Count (CBC) ▪ BUN, creatinine ▪ Electrolytes (including ionized calcium) ▪ Serum total protein, albumin ▪ Cholesterol, triglycerides ▪ CRP ▪ Coagulation (including ATIII) ▪ Immunoglobulins ▪ Complement (C3, C4)	▪ Urinalysis (early morning sample) ▪ 24-h proteinuria or Proteinuria/creatininuria (uP/uCr)
Additional	▪ Auto-immune markers (ANA, DS-DNA, ENA, ANCA) ▪ Thyroid function ▪ Infections (HBV, HCV, HIV, ParvoB19, CMV, EBV, pneumococcus, salmonella, treponema, mycoplasma…)	▪ Urine sodium

## Discharge and follow up

Patients can be discharged from hospital when proteinuria is falling and/or the following criteria have been fulfilled: stable clinical condition and body weight, no need for frequent biochemical testing, parents have been given instructions about the necessary follow-up care. Parents should be taught how to check for signs of relapse and how frequently to monitor their child’s urine at home.

- We suggest that, once discharged, patients should perform urine dipstick tests:
  - every other day during steroid tapering and during the first month after steroid withdrawal, then two/three times weekly.
  - daily, in the case of infection or positive stick.
  - immediately, in the case of edema

## Corticosteroid use for the first episode of INS

### Medication

The standard medication for the treatment of NS is oral prednisone (PDN) or its active metabolite, prednisolone. Prednisone has no substantial biological effects until it is converted to prednisolone via hepatic metabolism; Even though, in terms of their bioavailability, PDN and prednisolone cannot be considered equivalent they have been used indifferently at the same dosage both in clinical practice and in randomized controlled trials (RCTs), depending on the country. In the past, other corticosteroids (deflazacort, dexamethasone, bethametasone, methylprednisolone) [5–7] were occasionally used for the treatment of first episodes of pediatric steroid sensitive NS (SSNS), however, no RCTs have ever demonstrated their efficacy. In children with relapsing NS, a small single study showed that deflazacort maintained 66% more children with steroid dependent NS (SDNS) in remission during treatment, in comparison with PDN given in an equivalent dose [8], however, further RCTs of deflazacort are warranted in order to confirm its efficacy. Various steroids (deflazacort, dexamethasone, methylprednisolone), in association with immunosuppressive drugs, are used in place of PDN in steroid resistant subjects.

- PDN or prednisolone can be used indifferently for the treatment of a first episode of NS

### Dosing of prednisone

Prednisone doses of 60 mg/m2/day and 2 mg/kg/day are the internationally accepted standard doses for children with NS. In the 1970s, the members of the ISKDC agreed on an empirical dose of 60 mg/m2/day, followed by 40 mg/m2/day as the standard treatment for the first episode of INS. This agreement was based on the members’ personal clinical experience and a review of the literature. This dosing was also adopted by other study groups, such as the German Arbeitsgemeinschaft für Pädiatrische Nephrologie (APN) [9] and, more recently, the Haute Autorité de Santé (France) [10]. Alternatively, a PDN dose of 2 mg/kg/day was adopted in other countries (Canada, USA, India, etc.) (AAP, Indian Guidelines) because, in practice, it is easier to calculate. The 2012 Kidney Disease Improving Global Outcomes (KDIGO) guidelines recommend both dosages indifferently [4]. Recent studies have shown that the PDN dose of 2 mg/kg/day or 60 mg/m^2^/day cannot be considered equivalent for patients who weigh <30 kg [11]. Whether these different choices in PDN dosing have clinicl relevance is still a matter of debate [12, 13]; however, we decided to adopt the same dosage (body surface area dosing) for all our patients in order to avoid any hypothetical bias when evaluating the results of the therapy.

- We suggest that PDN be given at 60 mg/m^2^/day, with a maximum dose of 60 mg/day.

The optimal time to administer oral PDN treatment in order to maximise anti-inflammatory and immunosuppressive effects and minimize adverse events has yet to be clearly defined. The timing of PDN administration may influence the development of adrenal suppression, with morning and single administrations being potentially less suppressive than evening or divided-dose regimens [14], but definite improvements clearly need to be established in future clinical trials. Moreover, only one study (not an RCT) has focused on the correlation between timing of oral PDN intake and risk of side effects showing that PDN side effects (including hypertension) were less common in the single-daily-dose patients compared with the divided-dose patients [15].

In contrast, when comparing single-daily-dosing and multiple-daily-dosing patient groups, single dosing was as effective as multiple daily dosing in terms of maintaining remission in children who relapsed frequently [14]. Furthermore, in a recent update [16] of the Cochrane review, the authors state that during daily therapy, PDN is just as effective when administered in a single daily dose as it is when given in divided doses.

- We suggest that PDN can either be given in a single daily dose in the morning or in two divided doses (08.00 and 20.00).

### Treatment protocol

Since the first treatment regimen for an initial episode of NS was suggested by ISKDC in the 1970s, various treatment regimens have been used [17]. A Cochrane meta-analysis in 2000 concluded that a minimum of 3 months of PDN treatment resulted in fewer children relapsing by 12–24 months of follow up [18]. This is also reflected in the 2012 KDIGO guidelines, which recommend daily PDN treatment for 4–6 weeks, followed by alternate-day PDN dosing starting at 40 mg/m2 (or 1.5 mg/kg) for a total of 2–5 months, with tapering of the dose. More recently, an RCT has shown that PDN treatment (APN regimen) prolonged from 3 to 6 months without any increase in the cumulative dose did not benefit clinical outcome [19]. In 2015, the conclusions reached by the Cochrane systematic review were challenged by the results of the study carried out by Teeninga et al. and those of another two RCTs, which compared 3 months with 6 months of prednisolone treatment (cumulative dose 2792 vs 3530 mg/m2) [20] and 2 months with 6 months (cumulative dose 2240 vs 3885 mg/m2) [21], respectively. The authors did not find any difference in the time to first relapse or in the time to development of frequently relapsing NS (FRNS) between the 2 groups [22]. An update of the Cochrane systematic review in 2015 stated that when subgroup analyses according to risk of bias items are performed, there is no significant difference in the risk for FRNS between subjects treated with different steroid protocols [16]. In Italy, shared treatment guidelines were lacking and, as a result, the choice of steroid regimen and symptomatic treatments was based on the clinical expertise of each individual unit. A retrospective study evaluating the different therapeutic strategies adopted by pediatricians and pediatric nephrologists in a large number of Italian centers represented the first step towards establishing a shared protocol [23]. On the basis of the 2015 Cochrane systematic review, the KDIGO Guidelines and more recent data from the literature, we have adopted the following shared protocol (Table 5):

- We suggest that PDN (60 mg/m2, maximum 60 mg) be given daily in a single dose or divided into 2 doses for 6 weeks, followed by a single dose of 40 mg/m2, maximum 40 mg PDN on alternate days for another 6 weeks, without any tapering of the dose.

**Table 5:** Steroid protocol

Prednisone (PDN)	Dosage	Duration
Treatment of the first episode		
60 mg/m^2^ (maximum 60 mg)	in single or 2 divided doses	6 weeks
40 mg/m^2^ (maximum 40 mg)	on alternate days	6 weeks
Treatment of the first relapse		
60 mg/m^2^ (maximum 60 mg)	in a single or 2 divided doses	Until urine protein is negative for 5 days
40 mg/m^2^ (maximum 40 mg)	on alternate days	4 weeks

### First relapse

Approximately 80% of children with SSNS will relapse once or more. Of those, 50% will relapse frequently or become steroid dependent. We decided to establish a shared steroid protocol for the first relapse only, according to the treatment suggested by all the international guidelines [4, 10, 24, 25] (Table 5), while leaving therapeutic decisions for further relapses and for FR/SD subjects up to the clinical expertise of each individual unit.

- In the case of a first relapse, PDN should be given daily at a dose of 60 mg/m2/day (maximum 60 mg/day) in a single dose or divided into 2 doses, until urine protein has been negative 5 days. Thereafter, a single alternate-day dose of 40 mg/m2 (maximum 40 mg) should be continued for 4 weeks and then stopped.

## Diagnosis and treatment of edema in nephrotic syndrome

### Pathophysiology of edema in nephrotic syndrome

Edema is a predominant clinical feature of NS which occurs with variable severity, from moderate edema localized in particular areas of the body (face, legs, abdomen, genitals) to massive generalized edema [26]. There are two different mechanisms related to the pathogenesis of nephrotic edema. According to the underfill mechanism, the urinary loss of albumin leads to a decrease in plasma oncotic pressure which, with increased capillary ultrafiltration of sodium and water, leads to edema formation. Therefore, the retention of sodium chloride in NS could be a consequence of the activation of the renin-angiotensin-aldosterone system (RAAS) secondary to plasma volume reduction. In contrast, in the overfill mechanism the primary intra-renal sodium and water retention caused by resistance to atrial natriuretic peptide (ANP) and the activation of epithelial sodium channel (ENaC) in the inner medullary collecting duct leads to an expansion of the intravascular compartment and edema [27]. It is important to estimate the potential contribution of both underfilling and overfilling before starting diuretic therapy. Diuretic therapy alone is effective in “overfilled” patients in terms of the management of edema and intravascular volume excess [28]. On the contrary, the use of diuretics in patients with vascular “underfilling” could exacerbate intravascular hypovolemia. As suggested by some authors, intravascular volume can be easily evaluated by determining the fractional excretion of sodium (FENa), however, the FENa can also be influenced by salt and water intake and additional medication such as diuretics or ACE-inhibitors [29]. Kapur suggests that euvolemic patients with FENa >0.2% can only be safely and effectively treated with diuretics [30]. Hypovolemia may occur in patients with severe edema, or following the administration of diuretics in children with poor oral intake, diarrhoea, and vomiting. The clinical features of hypovolemia are abdominal pain, lethargy, dizziness and leg cramps, tachycardia, hypotension, delayed capillary refill, low volume pulses and cool, clammy distal extremities. An elevated blood urea nitrogen/creatinine ratio, rising hematocrit levels and low fractional excretion of sodium (<0.5%) suggest the presence of hypovolemia.

### Treatment of edema in nephrotic syndrome

The treatment of nephrotic edema in children, regardless of its severity, involves sodium restriction, diuretics and albumin infusions [31]. However, according to new clinical and experimental evidence, the most adequate therapeutic strategies are those which are adapted to each individual patient, thus permitting the optimization of the use of albumin and limiting the side effects of diuretics. For this reason the treatment of edema should be based on the severity of the clinical manifestations (Table 6).

**Table 6:** Management of edema

Management of edema in nephrotic syndrome	
Mild edema	▪ sodium restriction ▪ fluid restriction
Moderate edema	▪ sodium restriction ▪ fluid restriction ▪ loop diuretic ▪ potassium sparing diuretic for prolonged therapy
Severe/refractory edema	▪ sodium restriction ▪ fluid restriction ▪ loop diuretic +/− potassium sparing diuretic ▪ thyazide diuretic ▪ albumin, followed by a bolus of furosemide

#### Mild edema

Since treatment with corticosteroids usually leads to diuresis within 4–8 days, a mild edema (weight gain <7-10%) can be managed simply with dietary sodium restriction (<1-2 g/day or <35 mg/kg/day) and moderate fluid restriction (initial restriction of fluid intake to an equivalent volume of the patient’s insensible losses plus his/her urine output).

- We suggest that mild edema be managed with salt and fluid restriction only

#### Moderate edema

In patients with persistent edema and a weight gain of 7-10%, a loop diuretic such as oral furosemide (1–3 mg/kg/daily) is recommended, in addition to salt and water restriction. Additional treatment with potassium-sparing diuretics (e.g. spironolactone, 1–3 mg/kg daily) should be given to patients requiring higher doses and prolonged treatment with furosemide. Blood pressure should be monitored frequently and a gradual reduction of the edema over a period of one week is preferable. Diuretics should be avoided in patients with diarrhoea, vomiting, or hypovolemia.

- We suggest that moderate edema be treated with a loop diuretic, with the addition of a potassium-sparing diuretic in the case of prolonged therapy

#### Severe/refractory edema

In patients with a weight gain >10% and severe edema who do not respond to maximum doses of oral furosemide (and spironolactone), the co-administration of a thiazide diuretic (e.g. hydrochlorthiazide) may be indicated; furosemide might be better administered intravenously by bolus injections, under careful monitoring. For patients with refractory edema, infusions of albumin (20% albumin, 0.5–1 g/kg, over 3–4 h) with an i.v. bolus of furosemide (1 mg/kg intravenously) administered during or at the end of the infusion, are indicated. As the effect of these infusions is transient, patients with severe edema might require repeat infusions. All patients receiving albumin should be observed for respiratory distress, hypertension and congestive heart failure. Albumin should be administered with caution in patients with renal failure and is contraindicated in most patients with pulmonary edema. In these patients, acute hemodialysis with or without infusion of albumin should be considered.

- We suggest the co-administration of a thiazide diuretic in cases of severe edema unresponsive to oral or i.v. loop diuretics.
- We suggest albumin infusions in patients with severe edema unresponsive to oral or i.v. loop diuretics

#### Hypovolemia

If hypovolemia occurs, diuretic therapy should be stopped immediately. When signs of hypovolemia are absent, an increase in oral fluid intake alone may suffice. When the clinical signs of hypovolemia are evident, patients require hospital admission for prompt and careful correction.

- We suggest interrupting diuretic therapy immediately in case of hypovolemia and its prompt intravenous correction if clinical signs are present.

## Infections and immunization in children with INS

### Infections

Patients with NS, especially children, are susceptible to infection. Thanks to the use of antibiotics, the cumulative incidence of infection-related mortality has dropped from 40% to 1.5% [32], yet infections still represent one of the most frequent complications of NS worldwide. There are several predisposing factors of contracting an infection, such as the urinary loss of immunoglobulins, defective opsonization (reduced factor B and D concentrations), impaired T-lymphocyte function, the presence of edema and the effects of immunosuppressive therapy. Encapsulated organisms (mainly *Streptococcus pneumoniae*) and Gram-negative enteric organisms (predominantly *Escherichia coli*) are the most frequently involved; bacterial peritonitis is the most commonly occurring serious infection, with an incidence of 2-6% [1, 33]. Sepsis, meningitis, cellulitis and pneumonia may also occur and, although not severe, urinary tract infections and viral infections of the upper respiratory tract are common [34, 35]. Viral infections are generally well tolerated, with the exception of chickenpox, which can cause severe complications and be potentially lethal [36]. In the case of a severe viral infection, steroid therapy should be reduced or stopped.

### Prophylactic interventions

Much effort has been made to investigate the effectiveness of prophylactic interventions. In 2012, The Cochrane Collaboration reviewed 12 studies which had enrolled a total of 762 children with NS in order to assess the harms and benefits of the prophylactic interventions used for reducing the risk of infection in children and adults with NS [37]. The authors’ conclusions stated that the use of intravenous Immunoglobulin, thymosin, oral transfer factor, BCG vaccine injection, Chinese herbs (Huangqi granules and Tiaojining), may have positive effects on the prevention of infections in children with NS without causing severe adverse events. Unfortunately, due to the low methodological quality and the small number of RCTs, there is currently insufficient evidence for determining which of these interventions could be used for preventing infection in children with NS [37]. No RCTs on chemoprophylaxis have been performed in children with NS. The rationale behind the prophylactic use of penicillin was derived from studies in children with sickle cell disease [38]. Antibiotics have been suggested for younger patients with persistent anasarca, SRNS or FRNS, but there is little data supporting this practice and the risk of developing drug resistance casts doubts on its use.

- We do not recommend administering either i.v. immunoglobulin or prophylactic antibiotics to children.

The most effective intervention is early diagnosis. In the event of fever, children with NS should be examined as quickly as possible in order to initiate appropriate antibiotic treatment. Diagnostic and therapeutic decisions should be guided by the specific epidemiology of infections that usually affect NS children, as mentioned above [39].

- We suggest that rapid diagnosis and antibiotic treatment of infections are the most effective interventions.

### Varicella-zoster virus infections

Standard care in the United Kingdom and Australia includes post-exposure varicella-zoster immunoglobulin (VZIG), which is also recommended by the American Academy of Pediatrics (AAP) in immunocompromised children without evidence of varicella immunity (dose: 125U/10 kg, maximum 625U im) [40]. Despite VZIG administration, immunosuppressed and susceptible children may develop the disease with potentially fatal complications. An RCT showed how the prophylactic use of acyclovir (40 mg/kg/day divided in 4 doses for 7 days) may represent an effective additional measure, compared to VZIG only, in preventing the development of varicella in children receiving steroids [41].

- We suggest the use of oral acyclovir following exposure to chickenpox in non-immune patients.

Patients with a diagnosis of varicella should be started on acyclovir promptly (80 mg/kg/day divided into 4 doses for 5 days, with a maximum 800 mg/dose) in order to reduce the risk of visceral dissemination [36]. VZIG can be used either prophylactically or therapeutically, according to availability, epidemiology and local practice. In Italy, for instance, VZIG is not available.

#### Vaccines

Nephrotic syndrome is rare in children under one year of age, so the majority will have received most of their immunizations before clinical onset. Table 7 summarizes the safety of vaccines in children with NS. Patients on high-dose steroids should not be given live attenuated vaccines, while inactivated or ‘killed’ vaccines are considered safe [40]. Two studies have demonstrated the immunogenicity and safety of varicella vaccination in children with SSNS, but due to the limited size of the studied populations, no indications were provided regarding a possible increased risk of relapse (in Furth’s study, 6 out of 17 patients relapsed within 2 weeks of vaccination) [42, 43].

- We suggest that patients on high-dose steroids should not be given live attenuated vaccines.
- Once first episode treatment has been completed, the varicella vaccine is recommended three months after corticosteroid discontinuation.

**Table 7:** Immunization

Vaccine	Inactivated/Live, Attenuated	High dose steroids	Low dose steroids
Hepatitis B	I	YES	YES
Pertussis	I	YES	YES
Diphtheria	I	YES	YES
Tetanus	I	YES	YES
Polio (Salk)	I	YES	YES
H. Influenzae type B	I	YES	YES
Pneumococcal	I	YES	YES
Meningococcal	I	YES	YES
Flu	I	YES	YES
Human Papillomavirus	I	YES	YES
Varicella	LA	NO^a,b^	NO^c^
Measles	LA	NO^a,b^	NO^c^
Mumps	LA	NO^a,b^	NO^c^
Rubella	LA	NO^a,b^	NO^c^

^a^ Scottish Guidelines: feasible when high dose steroids (2 mg/kg/die for more than 7 days or 1,5 mg/kg/die for a month) have been discontinued for at least 3 months

^b^ AAP: feasible one month after high-dose (≥2 mg /kg/die, or ≥20 mg/day if the child weighs more than 10 kg) corticosteroids discontinuation if the patient has been treated for more than 14 days, or immediately after the discontinuation if the patient has been treated for less than 14 days

^c^ We recommend the use of live attenuated vaccines only after 3 months of corticosteroids discontinuation

Patients with NS have an increased susceptibility to severe infections due to encapsulated bacteria, especially *Streptococcus pneumonia*, because of impaired complement-dependent opsonisation. The universal pneumococcal vaccination of children under 2 years of age could ensure protective antibody titres to *S. pneumoniae* at the onset of disease [44]. The 23-valent pneumococcal polysaccharide vaccine (PPSV23) induces an adequate serological response independent of the time of vaccination (at disease onset, on high-dose steroid therapy or during remission) [45]; unfortunately, it does not effectively stimulate long-lasting immunity in children younger than 2 years of age [46, 47], in which a protective response follows the administration of the 7-valent pneumococcal conjugated vaccine (PCV7). For this reason, the AAP recommends vaccinating children under 2 years of age with PCV7, while PPSV23 should be administered after two years of age. For previously unimmunized children aged between 2 and 5 years, a priming dose of the conjugate vaccine should be followed by a dose of the PPSV23 8 weeks later. Revaccination after 5 years of age is considered for children (<10 years) with active NS. It remains to be said that no RCTs have been conducted in support of these recommendations [37].

- We suggest that all unimmunized children with NS should receive the pneumococcal vaccine.

## Thromboembolism in INS

Thromboembolic events in NS are classically described as a complication related to a combination of risk factors such as hypovolemia, hyperviscosity, urinary loss of anticoagulant factors, hyperlipidemia and thrombocytosis. Although these conditions are always concomitant in a clinical picture of full-blown nephrosis, thromboembolism is a rare complication in pediatrics; this means that for it to occur, other determining factors (genetic predisposition, infections, presence of central venous catheter, etc.) must be present. Even though the true incidence in children with INS is not precisely known, thromboembolic events are probably underestimated; they have been reported in 1.8-4.4% of patients with INS, while the percentage increases to up to 9% of patients in case studies including membranous, membranoproliferative and IgA nephropathies [48–51]. The highest rates of thromboembolic events (up to 25%) have been reported in children with congenital NS or secondary membranous or membranoproliferative nephropathies. Cerebral venous thromboses are more frequently observed (especially thrombosis of the sagittal sinus), followed by pulmonary thromboembolism, deep intracranial thrombosis and, less frequently, deep vein thrombosis of the lower limbs, the neck veins or peripheral arteries [52]. Clinically, the most common symptoms associated with thrombotic events localized in the brain are headache, altered mental state, papilledema, seizures and, rarer still, hemiparesis. Pulmonary embolism should be suspected in patients with pulmonary or cardiovascular symptoms, but many pulmonary emboli are silent in children with NS. Renal vein thrombosis is characterized clinically by the sudden onset of macroscopic hematuria, flank pain and/or tenderness. Some risk factors have been identified, such as degree of proteinuria, hypoalbuminemia, active infection as well as other factors such as thrombocytosis, anemia, hemoconcentration and hyperazotemia. Secondary thrombocytosis alone should not be considered a cause for alarm in children, because it is not necessarily associated with the occurrence of thrombotic events. This assessment would change, however, in the presence of predisposing conditions typical of the nephrotic condition, such as hyperviscosity [53] or hyperactive platelet function [54], both in adults and children with NS [55, 56]. When a workup for thrombophilia is performed on nephrotic patients with thromboembolic complications, coexisting genetic prothrombotic conditions (protein S deficiency, Antithrombin III (ATIII) deficiency, factor V Leiden, hyperhomocysteinemia and the presence of antiphospholipid antibodies) are frequently identified, suggesting the importance of this type of screening [52, 57]. Unfortunately, a workup for thrombophilia is not always performed in every patient who has developed thromboembolic complications, the same is also true for patients who have not developed a thrombotic event [57], therefore it is not possible to draw any definite conclusions as to just how important a test it is. In children with a history of severe protein C, protein S and ATIII deficiency, Lupus Anticoagulant (LAC) positivity or the presence of antiphospholipid antibodies, antiplatelet/anticoagulant prophylaxis can be considered, as well as for patients with a medical history of thrombotic events (Table 8). Recently, microparticles <1 μm in size with a prothrombotic function have been identified, which may derive from different cells (platelets, leukocytes or endothelial cells) and which have been found in increased numbers in children with NS [58, 59]. In conclusion, debate continues regarding the appropriateness of prophylactic anticoagulation to prevent NS-associated thromboembolism. This is due principally to the lack of any large, prospective randomized trials aimed at determining the efficacy and safety of such an approach. However, the matter raises concerns, given that most patients do not develop thromboembolism and therefore a significant number would receive prophylaxis unnecessarily. Therefore any potential adverse effects (i.e., anticoagulant-related bleeding) need to be carefully balanced against the expected benefit of thromboembolism prevention. An alternative approach would be to identify only those patients at highest risk for thromboembolism and target them for prophylactic therapy. This would potentially greatly diminish the number needed to treat to prevent a large proportion of thromboembolic events and avoid the therapeutic risks for those who are unlikely to develop thromboembolism [60–63]. In any case, thrombophilia screening should not be performed in the acute disease state, unless it is necessary to screen for genetic anomalies, as the urinary loss of antithrombotic factors can impede correct diagnosis.

- We do not suggest thrombophilia screening in children with INS at presentation unless there is a family history of thrombotic events at a young age (<50 years) or known abnormalities of pro-thrombotic coagulation factors.
- There are no indications for anticoagulant/antiplatelet prophylaxis in children with INS at presentation.
- We suggest the use of anticoagulant/antiplatelet prophylaxis in children with a concomitant cardiovascular abnormality (who would usually already be under prophylactic anticoagulant/antiplatelet treatment) or a central venous catheter (CVC) (almost impossible at the onset of illness).
- The greatest care must also be taken in patients with NS and a concomitant septic state, and/or in the presence of a CVC, for whom an antiplatelet/anticoagulant therapy should be considered.
- In cases of a persistent nephrotic condition in children with SRNS and whose edema is difficult to control, thrombophilia screening, as well as antiplatelet/anticoagulant prophylaxis may be considered.
- Secondary thrombocytosis usually does not require treatment. In the presence of active NS and a platelet count >1.000.000/mmc, we suggest aspirin prophylaxis.

**Table 8:** Thromboembolic events: therapy and prophylaxis

Drug	Indication	Dosage	Monitoring
Unfractionated heparin	Begin at the time of the acute event and continue for 5–10 days. Suspend on day 6 after OAT start, if INR on target. (Grad 1C +). Minor use in the last decade.	75 UI/kg bolus in10 min Initial maintenance dose: >1 year: 28 UI/Kg/h >1 year. 20 UI/Kg/h Then adjust to maintain aPTT between 60–85 s.	aPTT Therapeutic target: between 60–85 s.
Low molecular weight heparin (LMWH)	More used in the last decade in the treatment of thromboembolism in children	Enoxaparin Dosage (>2 months) Therapeutic: 100 UI/kg every 12 h Prophylactic: 50 UI/ kg every 12 h If clearance <60 ml/min) dosage must be adjusted on renal function	Anti Xa: blood samples 4 h after drug administration Therapeutic target: 0.5.1 UI/mL Prophylactic target: 0.3-0.5 UI/mL
Oral anticoagulants (warfarin)	Begin with heparin therapy until the target INR(2–3) is reached. Continue for 3 months, in absence of predisposing factors like NS. Continue for 6 months in presence of predisposing factors, like NS, or in cases of recurrent thrombosis. Vitamin K antagonists more used for older children (frequent blood check)	In pediatric patients > 10 Kg: 0.2 mk/Kg/day (For dosage adjustment, see Chest 2012 [61] and Paediatr Drugs 2015 [63]	INR Target: 2-3
Aspirin	If PLT >1.000.000 /mmc with concomitant NS	Empirical antiplatelet dosage in pediatrics: 1–5 mg/kg/day	
Fibrinolytic agents	No data on fibrinolytic treatment of thrombotic events in pediatric patients with NS. Use only in selected cases (urokinase, tPA) according to published recommendations [60, 61]		

For the therapy and prophylaxis of thromboembolic events we refer to the guidelines outlined in CHEST (2004–2012) [60, 61]

## Gastroprotection

The formation of ulcers during steroid therapy has been clearly demonstrated in experimental models, but the true incidence rate in humans is very low, especially in children. In recent times, no RCTs have been performed on this subject; however, the available data indicate an increased risk of gastric ulcers in the following situations: steroid treatment for over 4–6 weeks, cumulative doses >1000 mg and concomitant treatment with anti-inflammatory drugs and aspirin [64]. The real advantages of gastroprotection (and drug options) have not been adequately studied; the only systematic review in the literature concludes that there is no evidence strong enough to support the prophylactic use of proton pump inhibitors (PPIs) in oral steroid therapy in the absence of other risk factors for gastrotoxicity [65]. The prophylactic use of PPIs in children is therefore not indicated at NS onset, even in the case of high-dose steroids. Moreover, these drugs could worsen the risk of osteoporosis related to steroid treatment because calcium absorption in the stomach is reduced at an alkaline pH [66].

- We do not recommend the routine use of prophylactic PPIs in combination with steroid therapy in NS.
- We suggest that PPIs should be used only in selected cases manifesting with gastric symptoms resistant to treatment with malgadrate or alginate, or with any other risk factor (gastroesophageal reflux, esophageal disease, concomitant need for other gastrotoxic therapies).

## Supplementation with calcium and vitamin D

Steroids cause osteoporosis through the inhibition of osteoblasts and by increasing bone resorption [67] and the duration of steroid therapy and its cumulative dose correlate with bone density [68] moreover, low levels of 25-hydroxycholecalciferol [25(OH)D3] due to the urinary loss of both vitamin 25(OH)D3 itself and its binding protein can be noticed at onset and during relapses. Given the short duration of the relapse phase in SSNS, the effect on bone metabolism is minimal and still dependent on baseline levels of vitamin D, which vary in different populations as a result of nutritional status, sun exposure and other factors. No controlled studies are available; however, it is reasonable to conclude that, according to data from the literature, there is no need for vitamin D supplementation in steroid-sensitive forms in children from populations that do not have vitamin D deficiency (average levels above 20 ng/ml). Supplementation should be considered in frequent relapsers and populations with known vitamin D deficiency, where a reduction of lumbar bone mineral content was documented after short periods of disease activity and effectively prevented by supplementation with calcium and vitamin D [68]. Biphosphonates are seldom required in children, only in selected cases of persistent or SRNS, where supplementation with calcium and vitamin D is not sufficient [69].

- We do not suggest calcium and vitamin D supplementation in children at first episode or in SSNS unless vitamin D deficiency has been predicted or demonstrated.

## Treatment of hyperlipidemia

The majority of patients with NS or nephrotic-range proteinuria have hyperlipidemia [70], which can be explained by an increase in the hepatic synthesis of very low density lipoprotein (VLDL) and an accumulation of low density lipoprotein (LDL) correlated to the severity of hypoalbuminemia and proteinuria, leading to raised cholesterol levels similar to those seen in familial or congenital forms of dyslipidemia. In the severe forms of NS, an increase in triglycerides due to the reduced lipolysis of VLDL is also seen. In any case, in SSNS, dyslipidemia normalizes quickly after the remission of proteinuria and for this reason there are no indications for lipid-lowering treatments at the onset of NS, while the correction of dyslipidemia is recommended in steroid resistant subjects with persistent proteinuria in order to safeguard against the risk of atherosclerosis later in life.

- We do not recommend the use of lipid-lowering treatments at INS onset

In pediatric SRNS, some uncontrolled trials have demonstrated the safety and efficacy of statins and probucol in reducing cholesterol and triglycerides, yet the progression of renal insufficiency and proteinuria were unaffected, therefore the use of lipid-lowering drugs is not recommended in children [71]. A low fat diet has little effect if lipid consumption is not greatly restricted and therefore it is very difficult to use this approach with children. Supplementation with omega-3 can have a direct antiproteinuric effect and has a corrective effect on dyslipidemia, however there are no published data available recommending its use in children.

- We do not recommend low fat diets for children at INS onset

## Steroid Resistant Nephrotic Syndrome (SRNS)

Approximately 15-20% of subjects with NS fail to achieve complete remission after initial corticosteroid therapy and are classified as steroid resistant. The most important implication for these patients is that they are at significantly higher risk for the development of disease complications, as well as having a 50% increased risk of progressing to end-stage kidney disease within 5 years of diagnosis and a 30–50% chance of recurrence of the disease post-transplant [72]. The minimum requirement of corticosteroid exposure to define steroid resistance is still unclear. The ISKDC reports that 95% of children with SSNS achieve remission within the first 4 weeks of daily corticosteroid therapy and an additional 3% after a further 4 weeks [73, 74]. The KDIGO guidelines give a minimum exposure of 8 weeks of PDN 2 mg/kg/day (or 60 mg/m2/day) for 4 weeks, followed by 1.5 mg/kg (or 40 mg/m2) every other day for 4 weeks as their definition of resistance [25, 75]. Late remission after 8 weeks of steroid treatment has been demonstrated following prolonged exposure in low dose steroid therapy or following high-pulse doses in observational studies [76], but prolonged courses of daily corticosteroids are associated with an increased incidence of side effects.

- It is reasonable to define SRNS as a lack of remission despite 4 weeks of treatment with PDN at the dose of 60 mg/m2/day, followed by 3 high-pulse doses of Methylprednisolone (500 mg/m2) and another two weeks of PDN at the dose of 60 mg/m2/day

Children who were previously steroid sensitive but who developed persistent proteinuria after 4 or more weeks of corticosteroids following a period of remission are defined “late non-responders” and should be considered SR, too [72]. The most common histopathologic diagnosis in children with SRNS is focal segmental glomerulosclerosis (FSGS), followed by minimal-change disease (MCD), mesangial proliferative glomerulonephritis (MesPGN), diffuse mesangial sclerosis (DMS) and membranous nephropathy (MN) [77], although other histopathologic diagnoses are also seen. Treating these patients can be challenging and requires the expertise of a pediatric nephrologist. However, given the low probability (<5%) of achieving remission after 4 weeks of steroid treatment and given that steroids are ineffective in some histological pictures, it is reasonable to carry out a renal biopsy after 4 weeks of therapy; steroids can be continued for a further 2 weeks or immediately withdrawn, depending on the histological findings. When a child does not respond to steroid treatment:

- We suggest that kidney biopsy be performed after the first four weeks of therapy; the continuation of steroid treatment depends on the histological findings.

In recent years, abnormalities in a growing number of genes essential for podocyte development, structure and function have been identified in patients with congenital nephrotic syndrome (CNS) and SRNS, including podocin (NPHS2) and nephrin (NPHS1) and the advent of next generation sequencing will soon allow us to routinely screen all genes associated with SRNS [78]. In a large cohort of steroid resistant patients (1174 subjects) from The Podonet Registry, a genetic cause of the disease was identified in about 23%, the percentage decreases as the age of disease manifestation increases: from 66% in CNS to 15%–16% in schoolchildren and adolescents [77]. Nephrotic syndrome patients with mutations involving the abovementioned genes do not often respond to immunosuppressive therapy and have progressive kidney disease. For these reasons:

- We suggest that mutational analysis should be offered to patients with congenital, early onset (<12 m) NS or sporadic, familial SRNS or syndromes associated with NS

Extra-renal symptoms may be associated with these gene mutations (Table 9), more frequently alterations of the central nervous system (brain anomaly, microcephaly, and/or mental retardation); other features include symptoms suggestive of WT1 disease (sex reversal/urogenital abnormalities and cancer), impaired mitochondrial energy metabolism (myopathy, cardiomyopathy, and impaired hearing), Pierson syndrome (impaired vision), and Schimke syndrome (osteodysplasia).

**Table 9:** Genes associated with nephrotic syndrome

Gene	Inheritance	Characteristic signs and features
NPHS1	AR	CNS/NS
NPHS2	AR	CNS,NS - childhood and adult onset
CD2AP	?	Early-onset NS
PLCe1	AR	Early-onset NS
TRPC6	AD	Adult onset NS
PTPRO	AR	Childhood-onset NS
WT1	Sporadic; AD	Adult onset NS, Denys-Drash and Fraiser Syndromes
LMX1B	AR	Nail-Patella Syndrome/NS only
SMARCALI	AR	Schimke immuno-osseous dysplasia
E2F3	Chromosomal deletion	Early-onset NS and mental retardation
NXF5	X-linked recessive	NS with co-segregating heart block disorder
PAX2	AD	Adult onset NS
ACTN4	AD	Adult onset NS
MYH9	Risk allele	Adult onset NS
INF2	AD	Familial/sporadic NS
SYNPO	?	Adult onset NS
APOLI	Complex/AR	Adult onset NS
MYO1E	AR	Early or Adult onset NS
ARHGAP24	AD	Adult onset NS
ARHGDIA	AR	CNS
ANLN	AD	Adult onset NS
EMP2	AR	Childhood-onset NS
CUBN	AR	Intermittent nephritic range proteinuria and epilepsy
GPC5	Risk allele	Adult onset NS
PODXL	AD	Early or Adult onset NS
TTC21B	AR	NS with tubulointerstitial involvement
CLTA4	Risk allele	Sporadic NS
MTTL1	?	MELAS syndrome; NS+/− deafness and diabetes
tRNAlle	?	Deafness, NS, epilepsy, and dilated cardiomyopathy
tRNAAsn	?	Multiorgan failure and NS
tRNATyr	?	Mitochondrial cytopathy and NS
COQ2	AR	Mitochondrial disease/isolated nephropathy
COQ6	AR	NS with sensorineural deafness
ZMPSTE24	AR	Mandibulosacral dysplasia with NS
ADCK4	AR	NS
CYP11B2	Risk allele	NS, IgA nephropathy
LAMB2	AR	Pierson S.; CNS with ocular abnormalities; isolated early-onset NS
ITGB4	AR	NEP syndrome-NS, epidermolysis bullosa and pulmonary disease
ITGA3	AR	Epidermolysis bullosa and pyloric atresia + NS
LMNA	AD	Famlial partial lipodystrophy + NS
CD151	AR	NS, pretibilial bullous skin lesions, neurosensory deafness, bilateral lacrimal duct stenosis, nail dystrophy, thalassemia minor

*AR* autosomal recessive, *AD* autosomal dominant, *CNS* congenital nephrotic syndrome, *NS* nephrotic syndrome

## Indications for kidney biopsy

Indications for renal biopsy in children with NS are listed in Table 10 [79].

**Table 10:** Indications for renal biopsy in children with NS

Before treatment	• Onset at less than 12 months or more than 12 years of age
	• Initial macroscopic hematuria
	• Persistent hypertension and/or microscopic hematuria and/or low plasma C3
	• Secondary NS (Henoch-Schoenlein purpura, systemic lupus erythematosus, etc.)
	• NS associated with syndromes
	• Renal failure not related to hypovolemia
After treatment	• Steroid Resistance

Despite the absence of evidence-based recommendations regarding the role of renal biopsy in patients with SRNS, this procedure provides important information about renal histology and outcomes. Most patients with SSNS (90%) show MCD on renal histology. The renal histology in SRNS is different, with 30-40% of patients showing MCD, the same percentage showing FSGS and a smaller group, MesPGN [80]. Twenty glomeruli are needed in a biopsy specimen to confidently exclude lesions that affect only 5% of them, so in many routine biopsies containing fewer than this number it is possible to miss an FSGS lesion [81, 82]. Kidney biopsy will also provide information regarding the degree of interstitial and glomerular fibrosis, which will be utilized in the assessment of the prognosis of children with SRNS. Although light and immunofluorescence microscopy are the minimum requirement for the evaluation of histopathology specimens, electron microscopy helps to confirm the diagnosis of MCD and differentiate between primary and secondary FSGS, and it enables the diagnosis of early membranous nephropathy, membranoproliferative glomerulonephritis and Alport syndrome [73, 82]. Response to therapy may depend on renal histology. Patients with MCD show satisfactory response to therapy, while the presence of FSGS or chronic tubulointerstitial changes is associated with unsatisfactory outcomes [83]. A kidney biopsy may be useful in patients older than 12 years of age, considering the frequency of diagnoses other than MCD in this age group [25]. In fact, only 40-50% of teenagers with NS have an MCD [84–86].

- A renal biopsy should be recommended in patients <12 months or >12 years of age at the onset of NS or when secondary NS is suspected.

Differently, in African or African-American children, it is reasonable to perform the kidney biopsy at NS onset, before starting treatment. In fact, in these races the mean age for presentation of NS is higher than in Caucasians and Hispanics and there is a higher prevalence of FSGS or histological types different from MCD [4, 87–89]. However, the most important predictive factor of renal survival in pediatric NS is not the histological lesion but the achievement and the maintenance of remission after steroid therapy [90]. Kidney biopsy in children with FR- or SDNS is not required before initiating corticosteroid-sparing therapies because response to therapy is cited as the most important predictor of kidney survival.

## Conclusions

This consensus document is aimed at providing an updated, multidisciplinary overview on the diagnosis and treatment of pediatric NS at first presentation.

Until now, shared treatment guidelines were lacking in Italy and, consequently, the choice of steroid regimen was based on the clinical expertise of each individual unit. On the basis of a retrospective study evaluating the different therapeutic strategies adopted by pediatricians and pediatric nephrologists in a large number of Italian centers, the 2015 Cochrane systematic review, KDIGO Guidelines and a thorough review of the literature in the PubMed database, this working group (with the contribution of all the pediatric nephrology centres in Italy and on the behalf of the Italian Society of Pediatric Nephrology) has produced a shared steroid protocol that will be useful for National Health System hospitals and pediatricians.

It is the first consensus document of its kind to be produced by all the pediatric nephrology centres in Italy, in line with what is already present in other countries such as France, Germany and the USA. It is based on the current knowledge surrounding the symptomatic and steroid treatment of NS, with a view to providing the basis for a separate consensus document on the treatment of relapses and for future research.

## Abbreviations

[25(OH)D3]: 25-hydroxycholecalciferol
AAP: American Academy of Pediatrics
ANP: Atrial natriuretic peptide
APN: Arbeitsgemeinschaft für Pädiatrische Nephrologie
ATIII: Antithrombin III
CBC: Complete Blood Count
CNS: Congenital nephrotic syndrome
CVC: Central venous catheter
DMS: Diffuse mesangial sclerosis
ENaC: Sodium channel
FENa: Fractional excretion of sodium
FRNS: Frequently relapsing nephrotic syndrome
FSGS: Focal segmental glomerulosclerosis
INS: Idiopathic nephrotic syndrome
ISKDC: The International Study of Kidney Disease in Childhood
KDIGO: Kidney Disease Improving Global Outcomes
LDL: Low density lipoprotein
MCD: Minimal change disease
MesPGN: Mesangial proliferative glomerulonephritis
MN: Membranous nephropathy
NPHS1: Nephrin
NPHS2: Podocin
NS: Nephrotic syndrome
PCV7: 7-valent pneumococcal conjugated vaccine
PDN: Prednisone
PPIs: Proton pump inhibitors
PPSV23: 23-valent pneumococcal polysaccharide vaccine
RAAS: Renin-angiotensin-aldosterone system
RCTs: Randomized controlled trials
SDNS: Steroid dependent nephrotic syndrome
SSNS: Steroid sensitive nephrotic syndrome
VLDL: Very low density lipoprotein
VZIG: Varicella-zoster immunoglobulin
